# Perceptions, Knowledge, and Attitudes of General Population About Prostate Cancer-Associated Risk Factors: A Systematic Review of Qualitative Studies Focusing on Lifestyle

**DOI:** 10.1007/s11912-025-01653-7

**Published:** 2025-03-18

**Authors:** Catarina Leitão, Vanessa Neto, Luanna Silva, Marta Estrela, Margarida Fardilha, Fátima Roque, Maria Teresa Herdeiro

**Affiliations:** 1 Institute of Biomedicine (iBiMED), Department of Medical Sciences, University of Aveiro, Campus Universitário de Santiago, Aveiro, 3810-193 Portugal; 2 Federal University of São João del-Rei, Campus Centro-Oeste Dona Lindu, R. Sebastião Gonçalves Coelho, 400 - Chanadour, Divinópolis, MG 35501-296 Brazil; 3 Department of Social, Political and Territorial Sciences, University of Aveiro, Aveiro, Portugal; 4 Centre for Health Studies and Research, University of Coimbra, Coimbra, Portugal; 5 Health Sciences Research Centre, University of Beira Interior (CICS-UBI), Av. Infante D. Henrique, Covilhã, 6200-506 Portugal; 6 Biotechnology Research, Innovation, and Design for Health Products (BRIDGES), Research Laboratory on Epidemiology and Public Health Polytechnic of Guarda, Avenida Dr. Francisco Sá Carneiro, Guarda, 6300-559 Portugal

**Keywords:** General population, Lifestyle, Perceptions, Prostate cancer, Risk factors

## Abstract

**Purpose of Review:**

Prostate cancer (PCa) is the most prevalent cancer and the third deadliest in Europe among men. PCa has several well-established risk factors; however, the influence of lifestyle factors remains under investigation, which may hinder efforts to encourage healthier behavior adoption. Thus, this systematic review explored the general population’s perceptions, knowledge, and attitudes regarding PCa-related risk factors.

**Recent Findings:**

Eighteen qualitative studies were included after searching PubMed, Scopus, Web of Science, and EMBASE scientific databases between January 2013 and February 2023. Five major themes emerged from the 18 included studies: PCa knowledge, risk factors, lifestyle pattern changes, motivation/barriers to changing habits, and lifestyle advice support. Participants identified age, family history, genetics, and race/ethnicity as risk factors for PCa, but no consensus has been reached regarding lifestyle. However, most of the participants were willing to adopt healthier habits. Support from healthcare professionals (HPs), family, and friends, the desire for more time with loved ones, and fear of PCa consequences were cited as motivators for habit changes. However, poor economic conditions, work schedules, age, and PCa limitations hamper lifestyle changes.

**Summary:**

Effective interventions require personalized support and credible information from healthcare providers. Collaboration between family, friends, and HPs is crucial for promoting healthier behaviors and enhancing PCa management. This systematic review highlights the need for further research and innovative approaches to empower individuals towards healthier lifestyles, which could help prevent PCa or, at the very least, promote better treatment outcomes.

**Graphical Abstract:**

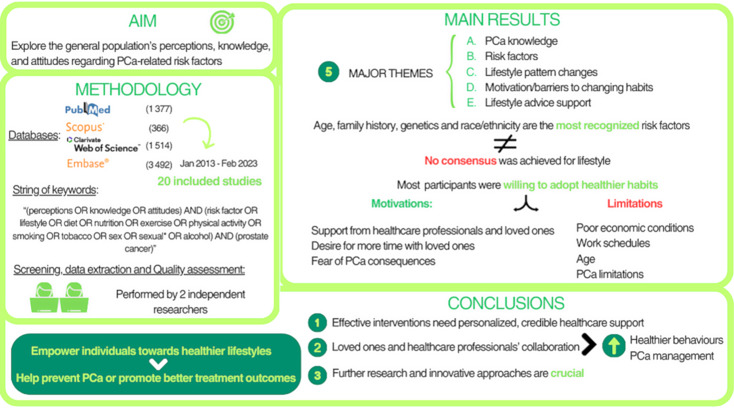

**Supplementary Information:**

The online version contains supplementary material available at 10.1007/s11912-025-01653-7.

## Background

Prostate cancer (PCa) is the most prevalent cancer affecting men in Europe [1]. The use of prostate-specific antigen (PSA) as a diagnostic tool has revolutionized the management and treatment of PCa, leading to a notable increase in its incidence across numerous countries. Nevertheless, PSA-based diagnosis can lead to overtreatment because it often detects slow-growing, clinically insignificant PCa that may never cause symptoms or affect a patient’s lifespan, yet still prompt aggressive treatments with potential side effects [2–4]. For these cases, active surveillance offers a viable alternative that can minimize unnecessary interventions and prioritize quality of life, as it involves regular PSA testing, digital rectal examination (DRE), imaging, and biopsies to monitor disease progression [5]. By postponing or potentially avoiding definitive treatment when there are no signs of progression, this approach reduces the risk of overtreatment and the unnecessary side effects associated with aggressive interventions [6–8]. Thus, it is imperative to devise novel strategies for precise diagnosis, which entails exploring potential risk factors that are yet to be fully understood.

Numerous risk factors have been linked to PCa, including advanced age, ethnicity, genetic alterations, and family history. However, emerging evidence indicates that adopting healthier lifestyles could enhance quality of life and improve prognosis [6, 9–14]. Despite this, many men are reluctant to alter their lifestyle habits post-diagnosis, largely due to a perceived lack of association between lifestyle choices and PCa or treatment outcomes [15–17]. Additionally, little is known about how lifestyle behaviors affect the likelihood of developing PCa. Recent research has highlighted the prevalence of abdominal fat, excessive body weight, and obesity [18] among men diagnosed with PCa, along with deficient diets lacking vegetables, nuts, and essential nutrients such as selenium, lycopene, and phytochemicals [19]. Given that nearly one-third of cancers are preventable through healthy dietary and lifestyle practices, PCa diagnosis presents an opportunity to educate individuals about the pivotal role of lifestyle factors in both the onset and progression of this disease.

Understanding the risk factors, especially those that are modifiable, and how the population receives and absorbs this information is important for political and health authorities to develop effective intervention strategies to promote a healthier lifestyle, both at the individual and collective levels [20]. It has often been reported that patients trust information and guidance from healthcare professionals (HPs). However, this communication is often lacking, leading to the unavailability of adopting measures for PCa prevention [21, 22]. Some systematic reviews have addressed the risk factors for the development of PCa [23–25]^,^ but none of them describes the perceptions of the general population.

To understand the perceptions, knowledge, and attitudes of the general population regarding the main risk factors associated with PCa, a systematic review of qualitative studies was conducted.

## Materials and Methods

### Protocol and Registration

This systematic review followed the PRISMA statement guidelines, and it was registered in the international database of prospectively registered systematic reviews (PROSPERO) (registration number CRD42023400342 [26].

### Search Strategy and Inclusion Criteria

For this systematic review, searches were conducted in the PubMed/MEDLINE, Scopus, Web of Science, and EMBASE scientific databases in February 2023.

The search was conducted by two independent researchers, and it was primarily designed to identify relevant studies on the perceptions of the general population about PCa risk factors, focusing on lifestyle factors. The following keywords and their equivalents were used in the scientific databases mentioned above:

(perceptions OR knowledge OR attitudes) AND (risk factor OR lifestyle OR diet OR nutrition OR exercise OR physical activity OR smoking OR tobacco OR sex OR sexual* OR alcohol) AND (prostate cancer).

The selection criteria applied in this review were as follows: (1) language: documents had to be published in English; (2) studies published from January 2013 to February 2023; (3) participants/population: adults/humans; and (4) types of studies that included qualitative studies (interview and/or focus groups). Study protocols, reviews, systematic reviews, and meta-analyses were excluded.

The included studies assessed the perceptions, knowledge, and attitudes of the general population regarding PCa-associated risk factors. All the studies that mentioned this impact were considered. Studies involving women were included because they provided insights into how women perceive PCa risk factors and their influence on male family members’ health awareness and decisions. Furthermore, while some included studies addressed cancers other than PCa, only findings related specifically to PCa were analyzed to maintain the focus of this review. All titles retrieved from the database were independently reviewed. The inclusion and exclusion criteria were applied by two independent researchers (C.L. and V.N.) and validated by a third researcher (M.E.) in cases where there was no consensus.

### Quality Assessment of the Included Studies

The quality of the included studies was assessed using the Critical Appraisal Skills Programme (CASP) Qualitative Studies Checklist [27], based on the study design of this systematic review. Like the screening process, the risk of bias in each study was evaluated independently by two researchers (V.N. and M.E.). Disagreements were resolved by a third researcher (C.L.).

### Data Extraction and Analysis

During study selection, Cohen’s Weighted Kappa analysis was conducted using IBM SPSS Statistics with two different timeframes to analyze whether C.L. and V.N. agreed. The first analysis was performed based on the title and abstract, and the second was based on full-text analysis.

The articles that met the inclusion criteria were summarized in a table for author and publication year, country, study design, setting, study population, sample size, participant characteristics, time frame, data collection procedure, themes explored, subthemes, and involved quotes. Two independent researchers (C.L. and V.N.) extracted data and compared their findings. In cases of disagreement, a third reviewer (M.E.) served as a referee to reach an agreement. After reading and analyzing the quotes from the included papers, LiquidText™ 2.7.50 software was used by two independent researchers (C.L. and V.N.) to assimilate the data and conduct a theoretical thematic analysis based on previously published literature [28–30]. Identified themes were derived from the data.

## Results

### Study Selection and Quality Assessment

**Fig. 1:**
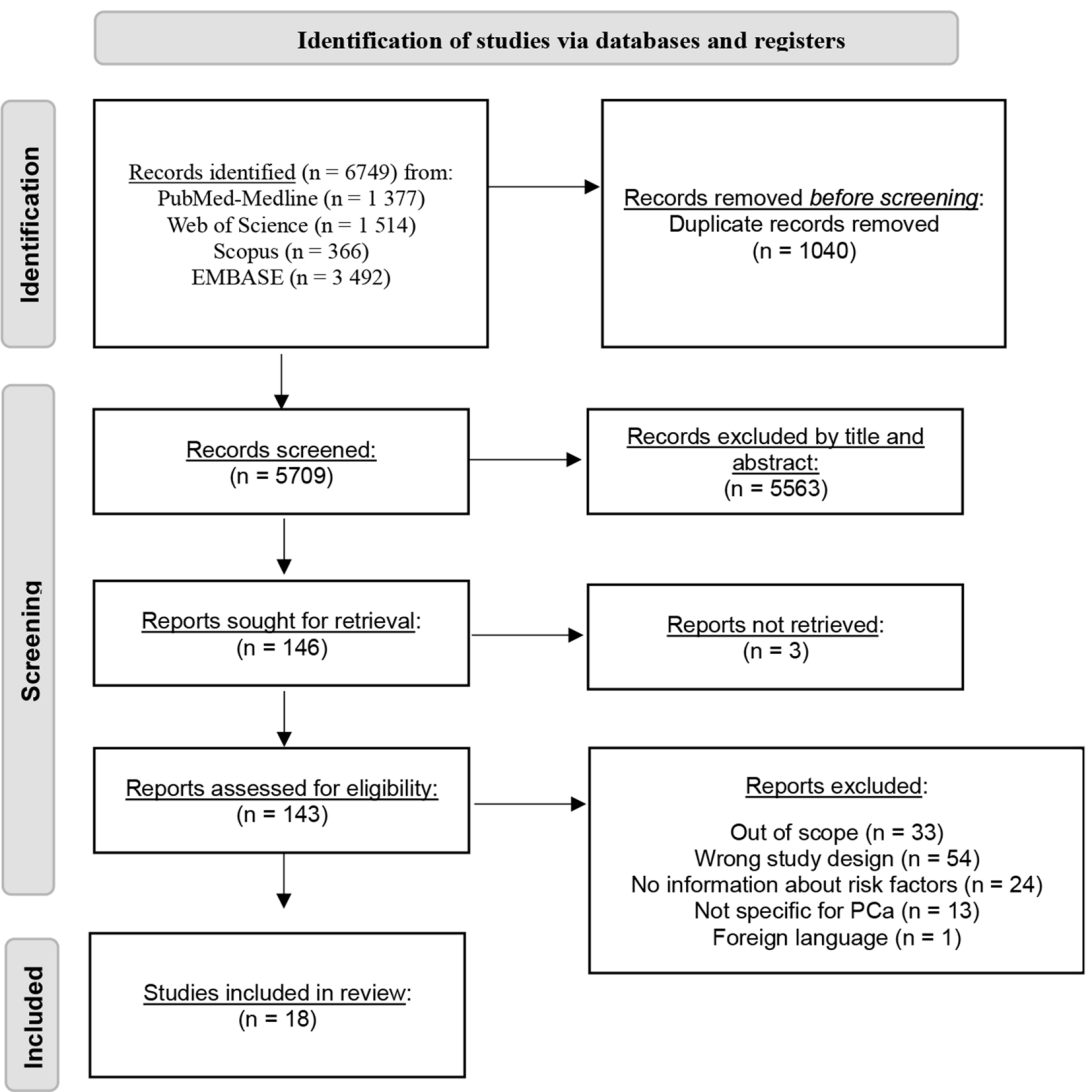
Application of search strategies to retrieve the total number of studies for analysis

The search strategy involved retrieving 6 749 citations from the PubMed–MEDLINE, Web of Science, Scopus, and EMBASE databases, from which 1 040 duplicates were excluded. Eligible articles were carefully chosen based on the title and abstract, resulting in 146 studies being analyzed. Since we couldn’t retrieve three of them, 143 studies were evaluated based on their full-text, of which 18 were deemed suitable for inclusion in the current review [31–48] (Fig. 1).

Cohen’s weighted kappa was assessed between the selection of studies based on title and abstract (Supplementary material, Figure S1) and full-text analysis (Supplementary material, Figure S2). For the selection based on the title and abstract, Cohen’s Weighted Kappa was 0.548, indicating a moderate level of agreement [49] between the two independent researchers. In the selection based on the full-text analysis, Cohen’s Weighted Kappa was 0.698, indicating a substantial level of agreement [49].

The results of the quality assessment (Supplementary Table S1) revealed that, overall, these qualitative studies exhibited a low risk of bias, considering that no section has demonstrated a significant concern for the results.

### Study Characteristics

The study characteristics of the included papers are summarized in Table 1, which provides information on the country, setting, study population, sample size, participants’ characteristics (age, gender, race), time frame, and data collection procedures.

**Table 1:** Characteristics of the selected studies

Authors (year)	Country	Setting	Study population	Sample Size (*N*)	Participants Characteristics	Time frame	Data collection Procedure
Bea et al., 2019 [31]	USA	Oncology clinic	Navajo cancer survivors	32	Age (A) = 56.9 ± 12.3 years old Gender (G) = 40.6% men and 59.4% women	May – Dec 2015	*Focus groups* + *interviews* to evaluate the perceptions of cancer causes, prevention, and treatment
Chien et al., 2022 [32]	Taiwan	Participants’ homes	Patients from the urological departments of 2 hospitals	13	A = 56–87 (mean age = 71.9) years old G = men	Aug 2017 – Jul 2018	*Face-to-face interviews* to understand the self-care experiences of PCa survivors before and while receiving androgen deprivation therapy (ADT)
Er et al., 2017 [33]	UK	Participants’ homes	African Caribbean men ≥ 18 years old with prostate cancer (PCa)	14	A = 52–80 (median age of 71.5) years old	2014–2015	*Semi-structured interviews* to evaluate the facilitators and barriers to dietary and lifestyle changes and the acceptability of a dietary and physical activity intervention
Ezenwankwo et al., 2021 [34]	Nigeria	Venues chosen by participants	Men diagnosed with PCa ≤ 2 years at the time of interview	27	A = 54–84 years old G = men	-	*Interviews*: (1) intrapersonal or individual level factors (knowledge of PCa, awareness of symptoms, risk factors/personal risk awareness, attitude towards screening), (2) interpersonal level factors (socioeconomic status, family, friends, social support), and (3) institutional/community level factors
Hicks et al., 2014 [35]	USA	Participants’ homes	Men who were treated for PCa and had at least one first-degree male relative (FDMR) and men who were FDMRs of a man treated for PCa	17	A = 25–43 years old G = men Race/Ethnicity (R/E) = 64% American Latinos	-	*Semi-structured interviews* and *follow-up interviews* to evaluate how familial communication about PCa risk and screening affects sons of men with PCa
Horwood et al., 2014 [36]	UK	Participants’ homes	Men without evidence of PCa but at elevated risk	21	A = 52–72 (65.4 ± 3.49) years old G = men R/E = Caucasian	Dec 2009 – May 2010	*Semi-structured interviews* to examine attitudes toward dietary modification for PCa prevention
Hunter et al., 2015 [37]	USA	Community health centers and churches	African American adults without a speech or hearing impairment	46	A = 24–72 (55 ± 15) years old G = 83% men R/E = African American	Apr – May 2012	*“Listening sessions”*: participants engaged in conversation with one another about their perceptions and beliefs regarding PCa risk and prostate-specific antigen (PSA) testing
Kassianos et al., 2015 [38]	UK	Telephone	Men who received a diagnosis of PCa	8	A = 55–76 (average age of 64,9) years old G = men	-	*Semi-structured interviews*: (1) perceived nature and importance of dietary change and (2) perceived determinants of dietary change
Keogh et al., 2013 [39]	Australia	-	Older PCa survivors	14 (8 non-ADT and 6 ADT)	A = 65.0 ± 6.5 (non-ADT), 65.8 ± 11.3 (ADT) G = men	-	*Focus Groups*: (i) perceived quality of life post-diagnosis, (ii) physical activity engagement post-diagnosis, (iii) perceived benefits, and (iv) perceived risks of physical activity
Malika et al., 2021 [40]	USA	-	Men ≥ 21 years old, who identified as African American, Caribbean immigrant, or African immigrant	51	A = 46–95 years old; R/E = 43% African Americans, 39.6% Caribbean and 17.4% African	-	*Focus groups*: risk, perspective on PCa education, screening, and PCa impact on sexuality) and key informant interviews
Mao et al., 2021 [41]	China	Quiet room	Patients with abnormal PSA index and/or digital rectal examination (DRE) and/or magnetic resonance imaging (MRI), who were advised by their doctors to receive a prostate needle biopsy (PNB) and had not received a PNB previously	30	A = 51–77 (average age of 67.2) years old G = men	Sep – Dec 2020	*One-on-one interviews*: (1) reasons to do the PNB, (2) symptoms, (3) their understanding of PNB, (4) reactions about the possibility of having PCa, (5) concerns about PNB and their disease, and (6) reason behind their disease
McIntosh et al., 2019 [42]	Australia	Telephone	Men fluent in English, that have been diagnosed with non-metastatic PCa, on androgen surveillance (AS) for at least 3 months, and not receiving palliative care, and their partners	18	A = average of 69.5 years old Partners’ age = average of 61.6 years G = 72% men	May – Aug 2017	*Interviews*: (1) attitudes and preferences towards exercise, and (2) attitudes and preferences towards exercise support
Menichetti et al., 2019 [43]	Italy	Hospital	PCa patients enrolled in the AS PRIAS protocol, patients who had been on AS for > 1 year, and patients that have been in AS for more (> 3 years) or less (1–3 years) time	24	A = 57–79 (average age of 68) years old G = men R/E = White	-	*Focus Groups* about perceptions of PCa patients on AS: (1) shared representations of views/meanings of health promotion, (2) barriers and facilitators for promoting health during AS, and (3) health promotion experiences and unmet needs
Nakandi et al., 2014 [44]	Uganda	-	Men > 18 years without any prior PCa diagnosis	545	A = 18–71 years old	Jul – Sep 2012	*Focus Groups* about PCa risk factors, signs, symptoms, and screening
Okoro et al., 2017 [45]	USA	Community center and the conference facility of a community-based organization	Black/African American men ≥ 18 years old	26	A = 30.9 ± 11.4 (W) and 35.4 ± 12.4 (M) years old; G = 59.9% women	-	*Focus Groups* to explore perceptions on the role of women in PCa prevention
Olapade-Olaopa et al., 2014 [46]	Nigeria	-	Participants ≥ 40 years	686 (29 FG and 656 Q)	A = average of 61.49 years old (FG) G = men	-	*Focus Groups*: (1) common causes of morbidity and mortality among men of their age group, (2) knowledge about the prostate, its diseases, and their perceived causes.
Robles et al., 2021 [47]	UK	Private research clinic room/ Telephone	Men diagnosed with localized PCa, undergoing prostatectomy	17	A = 53–81 years old G = men R/E = 94% White British/white and 6% Caribbean	-	*Interviews*: (1) causal beliefs about PCa, (2) perceptions of a healthy diet and PA before diagnosis, (3) barriers to adherence, facilitators of adherence, (4) and perceived benefits of behaviour change
Vapiwala et al., 2021 [48]	USA	Church	Participants ≥ 18 years who attended a large and diverse Centre City Philadelphia church with predominantly Latino and Black membership	34	A = 18–85 (average age of 52) years old G = 50% men R/E = 55.9% Hispanic/Latinos, 44.1% Blacks	-	*Focus Groups*: (1) general questions about health, such as current health practices, where respondents receive information about health, and when they visit healthcare professionals, and (2) questions specifically on PCa

#### Setting

Among all studies, six were conducted in North America [31, 35, 37, 40, 45, 48] and five (27.8%) in Europe [33, 36, 38, 43, 47] (Table 2). Three studies were conducted in Africa [34, 44, 46], two in Australia [39, 42], and two in Asia [32, 41].

**Table 2:** Distribution of the number of studies and their respective percentages by continent, setting, sample size, and data collection procedure

Study Characteristics	*n*	Percentage (%)
Total studies	18	100.0
*Gender*		
Conducted only in men	10	50.0
Conducted in both genders	10	50.0
*Continent*		
North America	6	33.3
Europe	5	27.8
Africa	3	15
Asia	2	10
Oceania	2	10
*Setting*		
Participants’ Homes/Venues chosen by them	5	25.0
Hospitals/Clinics	2	10.0
Community health centers/ churches	2	10.0
Community center and conference facility	1	5.0
Quiet room	1	5.0
*Sample size*		
< 20 participants	7	35.0
21–40 participants	10	50.0
> 40 participants	3	15.0
*Data collection procedure*		
In-person	13	72.2
Interviews	7	50.0
Focus Groups	5	33.3
Focus Groups + Interviews	1	6.7
“Listening sessions”	1	6.7
Telephone/Online	2	10.0
Interviews	2	100.0
In-person + Telephone/Online	1	5.6
Interview	1	100.0
Not Defined	2	10.0
Focus Groups	2	100.0

#### Sample Size and Study Population

Seven of the 18 included studies had a sample size of less than 20 participants [32, 33, 35, 38, 39, 42, 47], eight had a sample size of between 21 and 40 participants [31, 34, 36, 41, 43, 45, 48, 50] and three had more than 40 participants [37, 44, 46] (Table 2).

Almost all studies were based on age and gender to select participants (both genders and only men). Seven of the 18 studies included participants based on race/ethnicity [35–37, 40, 43, 47, 48]. All studies were conducted in adults, with most of which were carried out in people over 50 years old [32–34, 36, 38, 39, 41–43, 46, 47].

#### Data Collection Procedures

Most of the studies were conducted only in person [31–37, 40, 41, 43, 45, 46, 48] (Table 2). One study was conducted in a mixed setting [47] combining in-person and telephone/online, and two studies were conducted only by telephone [38, 42]. Among those conducted in person, five studies were conducted in participants’ homes or a venue chosen by them [32–36], three in clinics or hospitals [31, 43, 47], two in community health centers or churches [37, 48], one in a community center and conference facility [45] and one in a quiet room, with no further information provided [41].

Regarding data collection procedures, nine of the studies obtained data through interviews [32–36, 38, 41, 42, 47], while 7 collected data through focus groups [39, 40, 43–46, 48], one study through focus groups and interviews [31] and one of them through “listening sessions” [37] (Table 2).

#### Themes and Subthemes

**Fig. 2:**
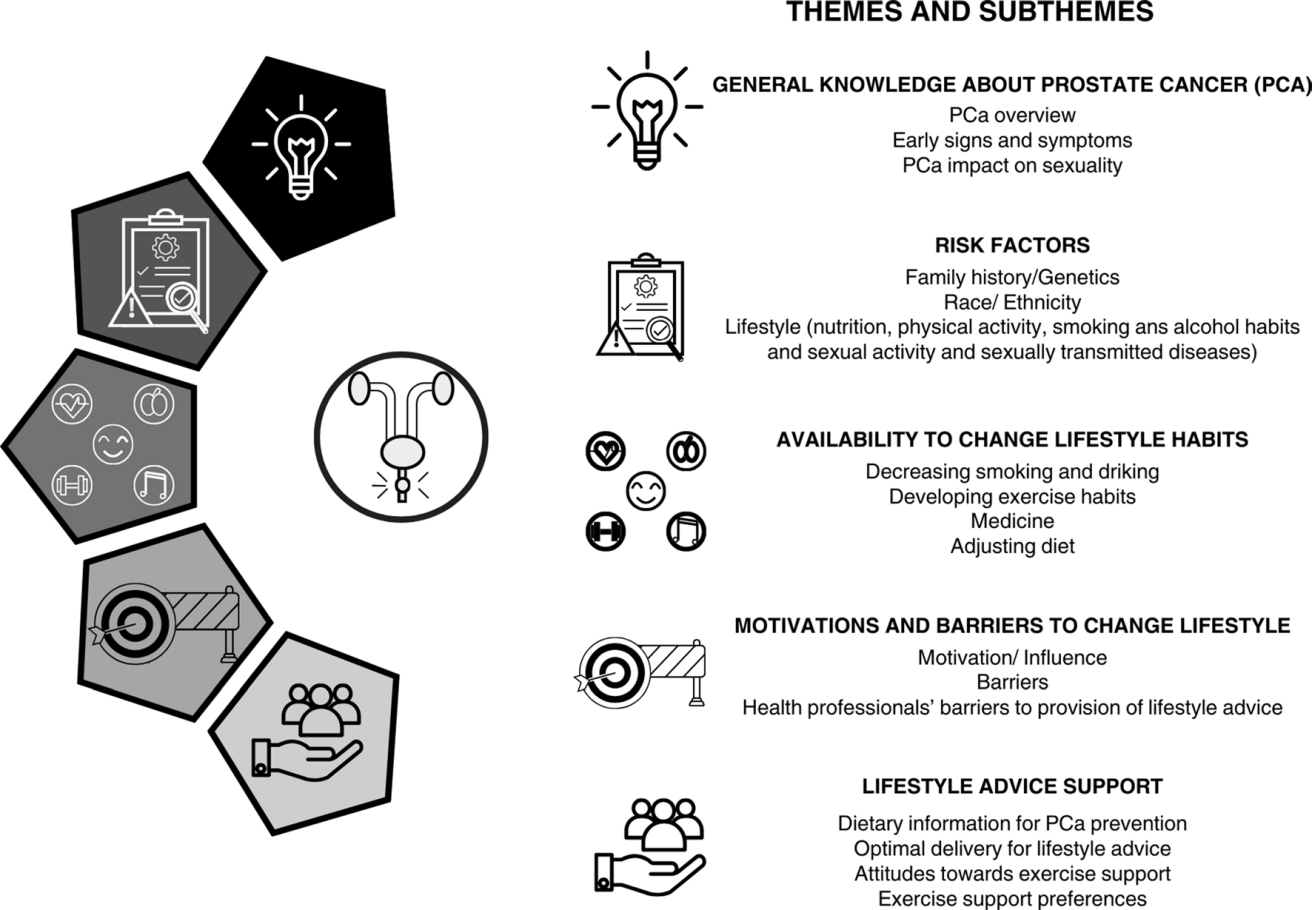
Themes and subthemes explored in the included studies

**Fig. 3:**
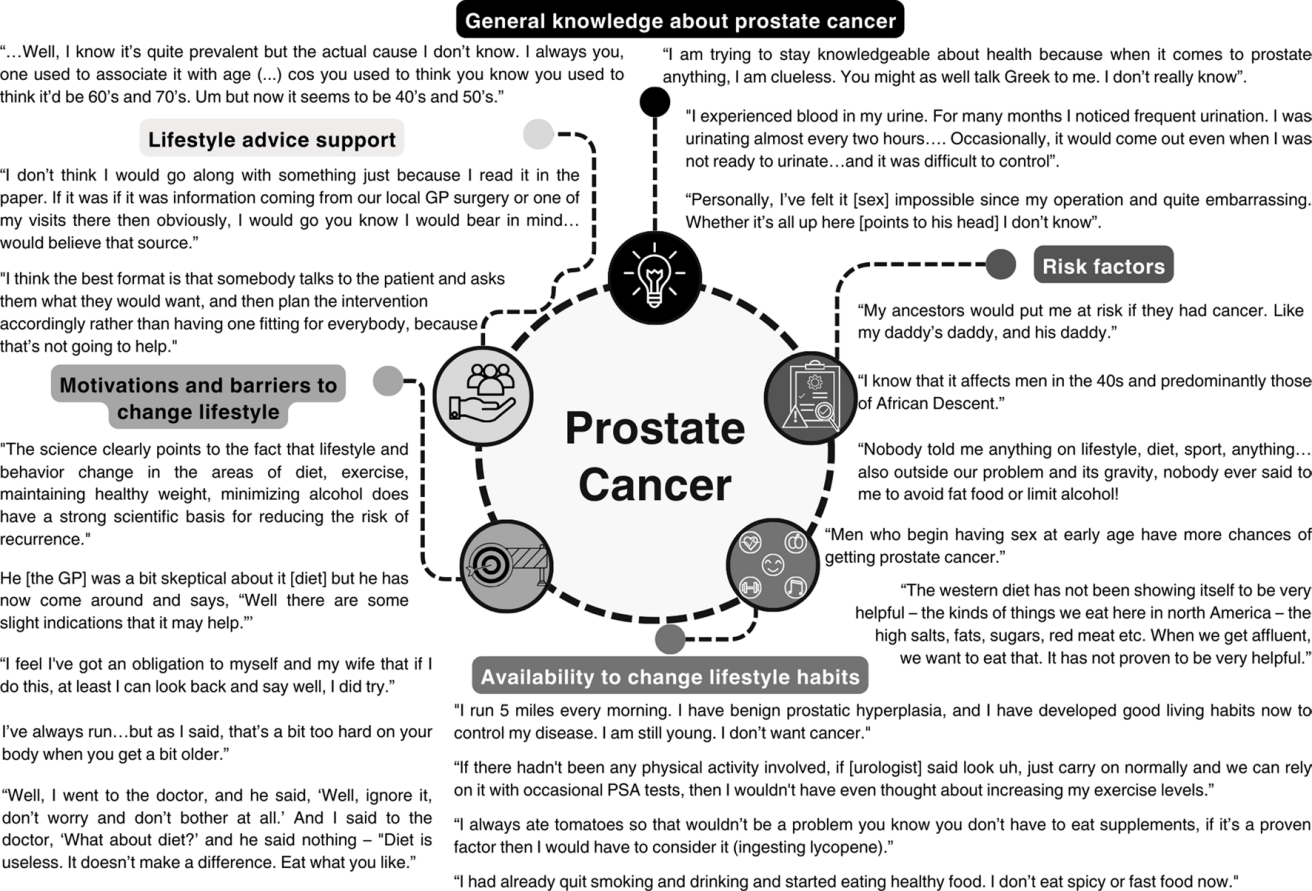
Representative quotes for each theme addressed in the included studies. Prostate cancer (PCa)

In this systematic review, five major themes emerged: general knowledge about prostate cancer; risk factors, lifestyle pattern alteration; motivation, and barriers to change lifestyle, and lifestyle advice support (Fig. 2 and for quotes examples see Fig. 3 and Table S2).

##### General Knowledge About Prostate Cancer

Regarding general knowledge of PCa, three subthemes arose: PCa overview, perceived symptoms, and the impact of PCa on sexuality. However, they established a correlation between age and PCa. Additionally, the impact of PCa on sexual performance was also a major concern for participants, as they described it as “embarrassing” and a sense of “losing masculinity” [39, 40, 48]. Moreover, this concern is also promoted by their knowledge of treatment side effects, as participants associated “taking the prostate out” with undergoing surgery and becoming impotent [40, 48].

##### Risk Factors

The risk factors’ theme included three subthemes, such as family history/genetics, race/ethnicity, and lifestyle factors (nutrition, physical activity, smoking, and alcohol habits, sexual activity, and sexually transmitted diseases), to which 12 studies contributed [31, 34–37, 40, 43–48].

Family history, genetic alterations, and race/ethnicity, mainly African Americans, have been identified as strong and well-established risk factors for PCa [31, 34, 35, 37, 40, 45, 46, 48]. Concerning lifestyle factors, there was no consensus among studies. While some of them reported that participants believed they had a healthy lifestyle, and for that reason, they could not develop PCa [34, 36, 40, 47], other studies have addressed the weak poor perception of a link between lifestyle and PCa development [36, 40]. Moreover, it was also noted that even among doctors, there was no encouragement for patients to alter their lifestyle habits [43].

##### Lifestyle Patterns Changes

Regarding lifestyle pattern changes, four subthemes emerged: decreasing smoking and drinking [32, 35], developing exercise habits [32, 33, 39, 41, 42, 47], use of traditional Chinese medicine [41], and adjusting the diet [32, 33, 35, 36, 38, 41, 47], to which nine studies contributed [32, 33, 35, 36, 38, 39, 41, 42, 47].

Almost every study reported participants’ willingness to adjust their diets [32, 33, 35, 36, 38, 41, 47], to become more active [32, 33, 39, 41, 42, 47], even if they were already active, and to decrease their smoking and alcohol habits [32, 35]. Some individuals have adopted stricter diets, including increased consumption of fruits and vegetables such as pumpkin and broccoli [32]; ingesting green tea or lycopene [36, 47], the latter present in tomatoes; and reducing red meat, dairy products, and sugar intakes [38]. Though, participants also recognized the challenge of maintaining strict long-term dietary alterations [38], which often leads to a gradual return to previous eating habits. This may be attributed to a lack of awareness about existing PCa-specific lifestyle guidelines and evidence-based recommendations [38, 43]. Participants mentioned being unfamiliar with any such guidelines, despite the availability of resources from organizations like the American Cancer Society [51]. These resources emphasize that adopting a healthy eating pattern, rich in fruits, vegetables, whole grains, and low in red and processed meats, sugar-sweetened beverages, and highly processed foods, may help reduce PCa risk [52].

##### Motivations/Barriers to Change Lifestyle

This theme included two subthemes: motivation/influence and barriers, of which seven studies contributed [33, 38, 39, 42, 43, 45, 47].

Concerning the motivation or influence of changing lifestyle habits, men feel there is a sense of obligation to oneself and loved ones [42] to make efforts towards health promotion, even if outcomes are uncertain, and family and friends [38, 47] provide support by encouraging and facilitating these adjustments. Regular exercise and changing diets have been emphasized for their physical and mental benefits, including a sense of accomplishment, a sense of control, and improved overall well-being [32, 39, 42, 47]. Additionally, general practitioners could play a significant role in promoting lifestyle changes, particularly when advising them to lose weight or to monitor their dietary habits [38].

On the other hand, there are also barriers to preventing individuals from altering their habits, such as busy work schedules [42], age-related physical limitations, physical discomfort in participating in certain activities [33, 42, 47], or skepticism [38, 43] regarding the effectiveness of altering their routines. A lack of clear guidance or support from HPs also emerged as a barrier, as some individuals reported receiving discouraging messages from doctors, such as dismissing the importance of diet changes [38].

##### Lifestyle Advice Support

The last theme encompassed four subthemes: (i) preferences for dietary information for PCa prevention, (ii) availability of credible dietary information influencing dietary actions, (iii) attitudes towards exercise support, and (iv) exercise support preferences, to which five studies contributed [36, 38–40, 42].

Regarding preferences for dietary information for PCa prevention, participants showed some skepticism about the reliability of information sources [36], particularly those from television and the Internet. They also expressed concerns regarding conflicting messages from scientific studies [36]. Participants highlighted the need for HPs’ instructions or advice about diet and physical activity before, during, and after treatment [38, 39], rather than from general media or external publications [36, 39]. Concerning attitudes towards exercise support, access to information about physical activity is crucial for choosing the best exercises and preventing abandonment [40]. Participants agreed that the benefits of exercise support positively influenced treatment outcomes and quality of life. Moreover, when asked about exercise support preferences, participants highlighted that when a specific exercise is recommended and supervised by a professional, especially for men with PCa, there is a higher chance of success and adherence [48].

## Discussion

This systematic review was conducted first to understand the general population’s knowledge about PCa risk factors and second, to comprehend whether men can adopt healthier lifestyles, including their motivations and barriers. The results of the studies analyzed in this systematic review show that it is necessary to improve awareness among society regarding the importance of avoiding unhealthy lifestyles. Despite lifestyle factors not traditionally being considered primary risk factors for PCa – due to the conflicting information available – this enhanced awareness may promote the adoption of healthier habits, improve quality of life, and potentially mitigate disease progression. While advanced age and family history are well-established risk factors, growing evidence supports the role of modifiable factors in influencing PCa risk and outcomes. This underscores the importance of educating the public on these associations and addressing misconceptions about risk factors to encourage proactive lifestyle changes.

### General Knowledge of Prostate cancer

Generally, participants of the included studies were unfamiliar with or had little knowledge about PCa and its symptoms. However, they correlated with advancing age. This is not surprising, as it is a well-established risk factor for this cancer [53]. A systematic review showed that the correlation between age and PCa was also perceived by young people [54]. This is a great concern for men, as they demonstrate feelings such as shame and disappointment for not being able to maintain their “masculinity” [55, 56]. Moreover, in addition to the fear of DRE and knowledge of PCa treatment side effects, inadequate health literacy leads to delays in screening, which could be a critical point for the treatment and monitoring of PCa [57–59].

### Risk Factors

Most participants were aware of the association between age, family history, genetic alterations, race/ethnicity (especially African Americans), and the development of PCa. Studies across various regions of the world have shown that such risk factors are recognized by both the general population and HPs [60–62]. Although many studies have demonstrated associations between lifestyle factors and PCa [63–65], participants in the reviewed studies often expressed uncertainty or a lack of awareness about these connections. In the past years, there has been an increasing number of studies reporting that an unhealthy lifestyle could promote its development and progression [63, 66–69]. In addition, lycopene has been shown to protect against PCa [70–72]. Nevertheless, there is a lot of conflicting information often disseminated through popular media channels (Internet, TV), and the inadequacy of the role of healthcare providers in raising awareness about PCa risk factors [73], as shown by differences among different countries [22]. Thus, receiving consistent information from HPs and understanding the benefits of physical activity and a balanced diet for this disease are important factors for adopting healthier habits, not only to prevent PCa but also to improve treatment outcomes and quality of life [74, 75].

### Lifestyle Patterns Changes

The adjustment of diet, engagement in physical activities, and reduction of harmful substances such as alcohol and tobacco have also been reported by other researchers. A study conducted in Italy showed that 43% of PCa patients changed their diet by reducing the consumption of red meat, processed meat, dairy products, and alcohol and sugary drink intake [76]. Bressi [74] pointed out that only 5% of patients diagnosed with PCa believed that physical exercise would not be beneficial, and 7.5% reported that it was inadequate or unsafe given their overall health conditions. Moreover, in a randomized controlled trial, following diagnosis, there was an overall reduction in alcohol intake and a reduction in the proportion of current smokers [77]. Despite the willingness to change dietary habits, it is important to emphasize that maintaining a very rigid diet is not sustainable in the long term [78]. Therefore, HPs could use the time of PCa diagnosis to raise awareness and promote healthier lifestyles in such a way that this change can be adapted to the normal life of men.

### Motivations/Barriers to Change Lifestyle

Regarding the motivations to change lifestyle, patients often view their sense of responsibility for spending more time and taking care of their families as a key motivation [42]. Moreover, family and friends support also play a critical role, both by encouraging changes and by fostering quality time during activities like exercise and food preparation [79]. Physical and mental benefits, such as a sense of accomplishment and improved self-image, further facilitate these changes [74, 75, 79]. Additionally, certain subgroups, such as younger men with families or those undergoing hormone therapy, may show higher motivation to adopt healthier lifestyles. As HPs noted, younger patients often embrace changes to ensure they can support their families, and the benefits of diet and exercise in reducing cancer recurrence provide further encouragement [80]. This aligns with participants’ recognition of the physical and mental benefits of healthier lifestyles and highlights the importance of integrating personalized advice from HPs into cancer care.

However, some barriers have been identified in adopting a healthier lifestyle. Lack of time and energy due to high workloads (often in multiple jobs due to low socioeconomic status) [42], as well as limitations imposed by age and PCa side effects such as urinary incontinence and physical discomfort [33], have been reported in other studies [81, 82]. A participant in the qualitative research conducted by Rana [75] reported, “Well, my exercise plan, I do not have time like I used to have, I am busy. I have got a heavy schedule”, corroborating our findings. Obstacles such as lack of resources and/or poor inconvenient locations for physical activity were overcome when participants found other alternatives, such as engaging in less structured activities (e.g., walking around the neighborhood instead of going to a gym), in previous studies [83, 84]. Regarding diet, many participants prioritize convenience over health (even though they possess culinary skills), often consuming fast food due to proximity to work, or opting for cheaper options in markets, sometimes comparing prices at more than multiple establishments [75, 79]. A lack of clear guidance from HPs emerged as a barrier to lifestyle change, with some participants reporting discouraging messages, such as dismissing dietary changes [38]. This aligns with the literature, where HPs recognize the benefits of diet and exercise but often feel it’s not their role to provide lifestyle advice due to time constraints and the patient’s condition [85] As one professional noted, “I don’t think it’s necessarily my role to provide generic lifestyle advice to everybody.”

### Support for Lifestyle Advice

Concerning support for lifestyle advice, information on lifestyle changes for PCa prevention is viewed skeptically by the patients. A good relationship between HPs and patients, along with simplified communication and educational materials, facilitates the adoption of healthier lifestyles. Patients reported low dissemination of information about PCa compared to other comorbidities such as breast cancer, systemic arterial hypertension, and diabetes mellitus [21, 22, 86]. Some countries like Australia have high levels of knowledge about PCa because of media campaigns transmitted and online commercials featuring respected celebrities [87]. A cohort study showed that the main source of knowledge about PCa was mass media (70.3%) [60, 88], demonstrating that TV and Internet resources can be beneficial, although they can be conflicting [73]. Furthermore, HPs also emphasize the importance of tailored advice to individual needs, rather than a one-size-fits-all approach, and that giving too much advice at once can be overwhelming, so focusing on one thing at a time works better. This is similar to what our participants said, as they preferred step-by-step guidance to make lifestyle changes easier [85].It is important to emphasize that HPs should consider patients’ lifestyle choices during decision-making. Participants pointed out that their preference for a particular physical activity was a crucial factor for long-term sustainability. Siapno et al. [79] showed similar statements to these results in their study. A systematic review elucidated that intrinsic motivation (the inherent pleasure derived from the behaviour itself) is the most predictive factor for long-term exercise adherence [89].

### Strengths and Limitations

This systematic review has several strengths, including the integration of a wide range of studies conducted across different continents, providing a global perspective on the understanding of PCa and its associated lifestyle factors. Additionally, employing a thematic analysis approach allowed for the identification of common themes across the included studies, providing a structured overview of the findings. By including qualitative data from interviews, focus groups, and listening sessions, this review also captured the nuanced perspectives and experiences of the participants regarding PCa and lifestyle habits. Additionally, to our knowledge, this is the first systematic review that includes qualitative studies focusing only on the perceptions of several risk factors and the availability to change lifestyle habits among the population with this type of cancer. Other similar systematic reviews that analyzed the perceptions among the population focused only on the views of African American men [25, 90], women [90], or other types of cancer [91]. However, this study also has some limitations, such as the limited scope of analysis, since it may overlook other potentially relevant factors that could influence behaviour change, such as socioeconomic status, cultural factors, and healthcare system characteristics.

### Implications for Clinical Practice and Research

This review underscores the critical need for healthcare providers to prioritize tailored, patient-centered communication about modifiable risk factors in prostate cancer (PCa). Clinicians should emphasize the importance of lifestyle changes in reducing risk and improving prognosis while addressing common barriers, such as lack of time and economic constraints. Moreover, the integration of psychosocial support, involving family and community, can enhance the adoption of healthier behaviors post-diagnosis. Future research should explore innovative interventions that bridge gaps in health literacy and foster collaboration between HPs, patients, and their support systems, ultimately improving PCa outcomes and quality of life.

### Conclusion

In conclusion, this systematic review highlights which risk factors are recognized by the target population for PCa, such as age, family history, and race/ethnicity, while noting the uncertainty regarding lifestyle influences. Despite this, participants in the included studies showed a willingness to adopt healthier habits, driven by a sense of responsibility and desire for quality time with family. However, barriers such as time constraints and socioeconomic factors hinder lifestyle changes. Effective interventions require tailored support and credible information from healthcare providers. Family, friends, and HPs also served as collaborators to promote healthier behaviors and improve PCa management globally, as shown in the motivation and barriers section. Further research and innovative approaches are needed to address these challenges and empower individuals towards healthier lifestyles.

## Key References

25. Coughlin SS, Vernon M, Klaassen Z, Tingen MS, Cortes JE. Knowledge of prostate cancer among African American men: A systematic review. Prostate. John Wiley and Sons Inc; 2021. p. 202–13.

- This systematic review examines knowledge of PCa among African American men, highlighting disparities in awareness and screening behaviors.

40. Malika N, Roberts L, Alemi Q, Casiano CA, Montgomery S. Ethnic Differences Among Black Men in Prostate Cancer Knowledge and Screening: a Mixed-Methods Study. J Racial Ethn Health Disparities. 2022;9:874–85.

- This mixed-methods study explores ethnic differences in PCa knowledge and screening among Black men, emphasizing the role of cultural and socioeconomic factors.

47. Robles LA, Shingler E, McGeagh L, Rowe E, Koupparis A, Bahl A, et al. Attitudes and adherence to changes in nutrition and physical activity following surgery for prostate cancer: A qualitative study. BMJ Open. 2022;12.

- This qualitative study investigates attitudes and adherence to lifestyle changes, such as nutrition and physical activity, following PCa surgery.

63. Leitão C, Matos B, Roque F, Herdeiro MT, Fardilha M. The Impact of Lifestyle on Prostate Cancer: A Road to the Discovery of New Biomarkers. J Clin Med. MDPI; 2022.

- This study analyzes the impact of lifestyle on PCa, highlighting the influence of dietary habits and physical activity on disease progression and the development of new biomarkers.

75. Bressi B, Iotti C, Cagliari M, Fugazzaro S, Cavuto S, Bergamaschi FAM, et al. Physical exercise habits, lifestyle behaviors, and motivation to change among men with prostate cancer: a cross-sectional study. Supportive Care in Cancer. 2022;30:5017–26.

- This study examines the relationship between nutrition, physical activity, and recovery after PCa treatment, emphasizing patients’ adherence to lifestyle change.

76. Rana B, Okere UC, Imm KR, Yang L, Housten AJ. Physical activity behaviour change in black prostate cancer survivors: a qualitative study using the Behaviour Change Wheel. Supportive Care in Cancer. 2024;32.

- This study explores barriers and facilitators to physical activity in Black PCa survivors, highlighting the role of capability, opportunity, and motivation in exercise adherence.

## Electronic Supplementary Material

Below is the link to the electronic supplementary material.

Supplementary Material 1

## Data Availability

No datasets were generated or analysed during the current study.
