# Supplementary material for: Perceptions, Knowledge, and Attitudes of General Population About Prostate Cancer-Associated Risk Factors: A Systematic Review of Qualitative Studies Focusing on Lifestyle
Source: Curr Oncol Rep. 2025 Mar 18;27(4):375–89. doi: 10.1007/s11912-025-01653-7 (PMC11976834; doi:10.1007/s11912-025-01653-7)
Supplement: Supplementary file 1 — Supplementary Material 1 [file 11912_2025_1653_MOESM1_ESM.docx]

**guidPerceptions, knowledge, and attitudes of general population about prostate cancer-associated risk factors: a systematic review of qualitative studies focusing on lifestyle**


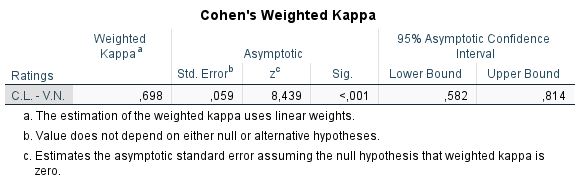


**Figure S2** - Cohen's Weighted Kappa for study's selection based on full-text analysis.


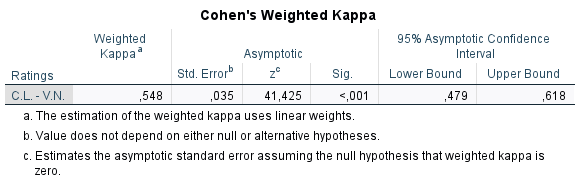


**Figure S1** - Cohen's Weighted Kappa for study's selection based on title and abstract.

**Table S1** - Quality assessment of the included studies.

| **Author (Year)** | **Section A** | | | | | | **Section B** | | | **Section C** |  |
| --- | --- | --- | --- | --- | --- | --- | --- | --- | --- | --- | --- |
|  | 1. Aims | 2. Methodology | 3. Design | 4. Recruitment | 5. Data collection | 6. Relationship | 7. Ethics | 8. Analysis | 9. Findings | 10. Value | |
| Bea et al., 2019 ^31^ | **Y** | **Y** | **Y** | **Y** | **Y** | **Y** | **Y** | **Y** | **Y** | **Y** | |
| Chien et al., 2022 ^32^ | **Y** | **Y** | **Y** | **Y** | **Y** | **Y** | **Y** | **Y** | **Y** | **Y** | |
| Er et al., 2017 ^33^ | **Y** | **Y** | **Y** | **Y** | **Y** | **Y** | **Y** | **Y** | **Y** | **Y** | |
| Ezenwankwo et al., 2021 ^34^ | **Y** | **Y** | **Y** | **Y** | **Y** | **Y** | **Y** | **Y** | **Y** | **Y** | |
| Hicks et al., 2014 ^35^ | **Y** | **Y** | **Y** | **Y** | **Y** | **Y** | **Y** | **Y** | **Y** | **Y** | |
| Horwood et al., 2014 ^36^ | **Y** | **Y** | **Y** | **Y** | **Y** | **Y** | **Y** | **Y** | **Y** | **Y** | |
| Hunter et al., 2015 ^37^ | **Y** | **Y** | **Y** | **Y** | **Y** | **Y** | **Y** | **Y** | **Y** | **Y** | |
| Kassianos et al., 2015 ^38^ | **Y** | **Y** | **Y** | **Y** | **Y** | **?** | **N** | **Y** | **Y** | **Y** | |
| Keogh et al., 2013 ^39^ | **Y** | **Y** | **Y** | **Y** | **Y** | **Y** | **Y** | **Y** | **Y** | **Y** | |
| Malika et al., 2021 ^40^ | **Y** | **Y** | **Y** | **Y** | **Y** | **Y** | **Y** | **Y** | **Y** | **Y** | |
| Mao et al., 2021 ^41^ | **Y** | **Y** | **Y** | **Y** | **Y** | **Y** | **Y** | **Y** | **Y** | **Y** | |
| Mclntosh et al., 2019 ^42^ | **Y** | **Y** | **Y** | **Y** | **Y** | **Y** | **Y** | **Y** | **Y** | **Y** | |
| Menichetti et al., 2019 ^43^ | **Y** | **Y** | **Y** | **Y** | **Y** | **Y** | **Y** | **Y** | **Y** | **Y** | |
| Nakandi et al., 2014 ^44^ | **Y** | **Y** | **Y** | **Y** | **Y** | **?** | **Y** | **Y** | **Y** | **Y** | |
| Okoro et al., 2017 ^45^ | **Y** | **Y** | **Y** | **Y** | **Y** | **Y** | **Y** | **Y** | **Y** | **Y** | |
| Olapade-Olaopa et al., 2014 ^46^ | **Y** | **Y** | **Y** | **Y** | **Y** | **Y** | **Y** | **N** | **Y** | **Y** | |
| Robles et al., 2021 ^47^ | **Y** | **Y** | **Y** | **Y** | **Y** | **Y** | **Y** | **Y** | **Y** | **Y** | |
| Vapiwala et al., 2021 ^48^ | **Y** | **Y** | **Y** | **Y** | **Y** | **Y** | **Y** | **Y** | **Y** | **Y** | |

Table S2 - Themes and respective quotes from the included studies.

| **Themes and sub-themes** | **Supporting quotes** | **Contributing studies** |
| --- | --- | --- |
| General knowledge of PCa | | |
| PCa overview | “…Well, I know it’s quite prevalent but the actual cause I don’t know. I always you, one used to associate it with age…but now you know cases are coming to light where men are getting it, appear to you know it’s it’s more publicized that they’re getting it at an earlier age cos you used to think you know you used to think it’d be 60’s and 70’s. Um but now it seems to be 40’s and 50’s you know sort of particularly things like you get publicity about sportsmen and things like that er getting it”. | ^36,38,40,44,46^ |
|  | “I was talking with someone who said that diet and some of these things are good to be attentive to. He also discussed sexuality and talked about the different approaches to sex and the timing and those things; something about the frequency of sex associated with better prostate health. He in fact observed that he and his wife are applying these finding to their lives”. |  |
|  | “I don’t really know exactly what it’s about but I’ve heard of it [PCa] but I’ve never met anyone that has it. I just know it’s a disease as simple as that. Cancer, once you hear cancer, you that that person is going to go, unless God intervenes”. |  |
|  | “I am trying to stay knowledgeable about health because when it comes to prostate anything, I am clueless. You might as well talk Greek to me. I don’t really know”. |  |
| Perceived symptoms | "I knew nothing about [the disease] until it was said [I] had it. I was only experiencing symptoms I did not like. I went to the hospital to discuss it… From there I began to undergo a series of tests until they discovered it. [The doctors] said it was [PCa]. I did not know what it was." | ^34,35,46^ |
|  | "Three years plus. I never considered [them] as something serious. I am hypertensive and had linked those symptoms to hypertension when I first saw them." |  |
|  | "I experienced blood in my urine. For many months I noticed frequent urination. I was urinating almost every two hours…. Occasionally, it would come out even when I was not ready to urinate…and it was difficult to control”. |  |
|  | "I thought it was due to old age and nothing serious…". |  |
| Impact of PCa on sexuality | “I heard it in another church, they were saying ‘don’t let them take your prostate out, you won’t be able to’ …you know.” | ^35,39,40,48^ |
|  | “Everyone wants to be Mr. Man. I’m Mr. Player. Nobody’s gonna talk about nothin’ like that. That’s a whole thing on your manhood”. |  |
|  | “I just don’t worry about it [sex] now. My wife just sort of accepts it, and we just get on with life. It’s not there and that’s it.” |  |
|  | “Personally, I’ve felt it [sex] impossible since my operation and quite embarrassing. Whether it’s all up here [points to his head] I don’t know”. |  |
|  | “There is no interest, no libido.” |  |
|  | “I have a friend who had that experience of being diagnosed with prostate cancer and he was struggling for years with weight. He decided to go ahead and have the surgery and he is impotent now. I guess they cut a nerve of some kind that would have allowed him to maintain his sexuality”. |  |
|  | “If you have had prostate cancer it doesn’t mean that your sexuality is gone. If you check and get early diagnosis you will have a chance. A lot of people do not know that if it is detected early your chances of getting rid of it are great”. |  |
| Risk factors | | |
| Family history/Genetics | “We went to Mexico, and every time [extended family members] call the house, and they told me that they had it, and they turned out [okay]. All these stories kind of pop up as soon as someone focuses [on PCa] . . . all these stories start popping up, from my uncle and family members”. | ^27,34,35,40,45,46^ |
|  | “My sons are well educated on prostate cancer but that’s only because their father had prostate cancer. Once he got diagnosed, we as a family had to learn, otherwise I don’t think they would have known much about it”. |  |
|  | “Family history plays a big factor in getting this disease but that is what disadvantages Africans. If when someone dies in the village there is no way to know cause of death and so when we ask people their family history, they say there is no way for them to know. But that is important to understand their risk”. |  |
|  | “When first reflected on my health, I knew I was at increased risk for prostate cancer since my father had prostate cancer. I remember I was turning 40 and I knew it’s about that time to start to get tested”. |  |
|  | “…there can be some genetic things that even obviously be passed down from your family but um overall you get the feeling that it is more to do with your lifestyle and the way that perhaps abuse your body with various things that can bring on some of these cancers, but that’s what the papers say you know I just don’t know”. |  |
|  | "My ancestors would put me at risk if they had cancer. Like my daddy’s daddy, and his daddy". |  |
| Race/Ethnicity | “I know that it affects men in the 40s and predominantly those of African Descent”. | ^37,40,46,48^ |
|  | “When I came to America the first time, I never knew anybody who was diagnosed with prostate cancer. I never knew of anybody who died of prostate cancer. But when I came to America as the years go by, I came to see that it’s a widespread thing among black men. I came to think that. it’s a culture thing because before we never knew about this when we came to America, and we never experienced it in the Caribbean. But I am in America now and its part of the American culture”. |  |
| Lifestyle (nutrition, physical activity, smoking and alcohol habits and sexual activity and sexually transmitted diseases) | "I did not think of myself as one [who could develop PCa] because…I do not drink alcohol and I do not smoke. So, I never thought I could develop PCa". | ^34,36,40,43,44,46-48^ |
|  | “For some cancers yes like lung cancer you don’t smoke um, but I think uh there are a lot of rumors and myths if you like about certain diets um preventing or helping to prevent certain types of cancer”. |  |
|  | “I imagine keeping you fit I can’t imagine that they would in as such prevent cancer other than they keep you fit and then if you are fit you might then fight off things but other than that, but as a preventative I’m not sure about that”. |  |
|  | “Nobody said me anything on lifestyle, diet, sport, anything…also outside our problem and its gravity, nobody ever said to me to avoid fat food or limit alcohol! I'd like to know from doctors if there is a list of things to certainly avoid so to limit dangers”. |  |
|  | "I really, sort of, eat a fairly Mediterranean diet. I use olive oil instead of butter, for example. If I have a sandwich or something, I put olive oil on. We cook all our own vegetables. I used to have an allotment, which I had to give up because of my leg, because of my knee”. |  |
|  | "I tend to do stretching exercises every day and I do a lot of gardening as well. I love gardening and I do walk. As I say, I’ve got two little terriers”. |  |
|  | “I invited a particular brother to come to this focus group and he said he is not coming because they are talking foolishness there. The only reason we have more prostate cancer is because we do not eat enough pasta. And he goes, Italians do not have a high rate of that disease because they eat a lot of pasta”. |  |
|  | “When you have sex with a woman who has “candida”, she can put that “candida” dirt in you, and it could end up causing cancer.” |  |
|  | “Men who begin having sex at early age have more chances of getting prostate cancer.” |  |
|  | The western diet has not been showing itself to be very helpful – the kinds of things we eat here in north America – the high salts, fats, sugars, red meat etc. When we get affluent, we want to eat that. It has not proven to be very helpful”. |  |
| Willingness to change lifestyle habits | | |
| Decreasing smoking and drinking | “I am reducing my alcohol consumption slowly.” | ^32,35^ |
|  | “I will slowly reduce my tobacco consumption…I was inspired and impressed by my grandson…He said, ‘I hope grandpa does not smoke.'" |  |
| Developing exercise habits | “I use my cell phone to catch Pokémon to encourage me to walk.” | ^32,33,39,41,42,47^ |
|  | “Walking makes my mood better and sleep better.” |  |
|  | "I run 5 miles every morning. I have benign prostatic hyperplasia, and I have developed good living habits now to control my disease. I am still young. I don’t want cancer." |  |
|  | “Look I'm reasonably active, I'm mobile, I've got quite a large ah, garden. I do a lot of gardening … I go fishing ah quite regularly … bit of woodworking in the shed, and so forth.” |  |
|  | “If there hadn't been any physical activity involved, if [urologist] said look uh, just carry on normally and we can rely on it with occasional PSA tests, then I wouldn't have even thought about increasing my exercise levels … so it was because of the recommendation of the gym program that caused me to be aware of increasing exercise” |  |
|  | "…I built my walk into the gym routine. I did 30 minutes on a treadmill sitting at about 6 kph or something like that with grading”. |  |
|  | "I think, I mean, although I’ve painted a picture of being quite active, then you know, I mean, it wasn’t very organized, you know what I mean? What this did was to impose a routine on me, which I was quite happy with. And it’s like setting a goal, isn’t it?” |  |
|  | "The walking because it kept me a bit healthier and fitter, I think I did better on the radiotherapy.” |  |
|  | “I swim, I walk…generally keep fit and active”. |  |
|  | “I have increased. I used to probably walk two maybe three times a week before. As a consequence of the prostate problem, I decided I would walk a bit more and so keep fit.” |  |
|  | “I started going to the gym twice a week, for about an hour each time. I feel better after that.” |  |
|  | “Exercise [weight training] is really good, and its frustrating as a guy to lose muscle mass.” |  |
|  | “Well, I used to play every Sunday…but since I had the operation, I haven’t played…when I am feeling healthy and okay I will.” |  |
|  | “I play golf twice a week, I go to the gym twice a week, I try to do some stretches…I walk every day.” |  |
| Medicine | “I have seen a superior Chinese doctor who prescribed Chinese medicine for me. It is useful and I take the medicine three times a week.” | ^41^ |
|  | “I don’t take Western medicine. I eat pollen. I learned from a health program on TV that it is a type of traditional Chinese medicine and is used as a prostate medicine. It is effectively controlling my urinary disease.” |  |
| Adjusting diet | “I already ate [like that] for 3 years! Ate pumpkin and broccoli for breakfast…” | ^32,33,35,37,38,41,47^ |
|  | "It [diet] is more important. Although I am stricter in my diet, I think actually that I enjoy food more now. It just used to be something you did three times a day – you put the fuel in. Nowadays because I choose and I watch what I eat, I think it is true to say that I enjoy food more now. We can take more care over the preparation of food.” |  |
|  | "It [the diagnosis] made me more aware of certain things I should definitely be avoiding. . .reduction in red meat. . .reduction of dairy products and reduction in sugar." |  |
|  | "The doctor told me it may be PCa. I had already quit smoking and drinking and started eating healthy food. I don’t eat spicy or fast food now." |  |
|  | “I don’t see why not (green tea). Very very strong chemicals come from nature, and I think we are using them all the time aren’t we, we just don’t realise it? The pharmaceutical companies are supplying them, storing them, and testing them by the millions aren’t they from nature just trying to find effective drugs and things, so there is no reason why it shouldn’t come from there.” |  |
|  | “If we heard it was beneficial and it was proved to be so then it’s one of the easiest things in the world to just switch to drinking green tea.” |  |
|  | “I always ate tomatoes so so that wouldn’t be a problem you know you don’t have to eat supplements, if it’s a proven factor then I would have to consider it (ingesting lycopene).” |  |
|  | "It becomes very easy, because the Lycopene, I took every morning with my hypertension medication, and it just became part of the breakfast.” |  |
|  | “…But since I was diagnosed, er, we just, I would say about, the beef probably come out, we cut down beef a bit, you know? … they always say too much red beef is not good for you and that. So we just cut it down, you know?” |  |
|  | “She [dietician] said with, with the prostate cancer that I’ve got I should eat a lot of tomato. Tomato is good for the prostate cancer. My doctor before, Doctor M, told me that if I drink pomegranate juice it’s a little bit helpful as well…That’s what I buy, we buy, things, we buy pomegranate juice.” |  |
|  | “It would be easier, but- but, I mean, it’s- yeah, it would be easier, without a doubt. If you could take a tomato tablet that, does it, but then it’s what goes into making the tablet. With a tomato, a fresh tomato, you know what you’re getting, don’t you?” |  |
| Motivation and barriers to change lifestyle | | |
| Motivation/Influence | "My wife was trained in domestic science, and she has, broadly speaking, kept me on a healthy diet with a good variety and plenty of fruit and vegetables." | ^33,38,39,42,43,45,47^ |
|  | "Our children grew up and left home and [when they lived at home] they all wanted meat and dairy and all sorts of things which I didn’t want to eat. . .Now when they come and stay with us I don’t go and cook the things they want and probably join me eating as well. [Family] circumstances made it easier for me to control my diet [post-diagnosis] and so I chose to go a certain way." |  |
|  | “When I finish the gym work I have a hot shower, I'm re-invigorated, invigorated, I feel good…you can jump under the shower and feel you've accomplished something.” |  |
|  | “I feel I've got an obligation to myself and my wife that if I do this, at least I can look back and say well, I did try. If it doesn't work at the end of the day and something goes wrong, I can't…lay in bed and say well, if I had done this or done that, that maybe it would have been different?”. |  |
|  | “I think the exercise, uh, kind of stimulated him into thinking, ‘look, I can do more than this’ … I think he thought it was going to help him, that it was in his interests to, to pursue it.” |  |
|  | "She is wonderful and she looks after me absolutely, 100%, our food is ready by six…My wife is a three veg, four veg, five veg and, she is greens, she thinks they are wonderful.” |  |
|  | ‘For me there is a clear benefit, exercise and keeping fit is the difference between black and white. Both for health purposes and physical well-being.’ |  |
|  | ‘I feel good [after physical activity]. I do feel good in a holistic thing. That’s why I try and do it in the morning. Because you try and bank it. It does sort of set me up for the day.’ |  |
|  | "He [the General Practitioner] said, ‘It would be helpful if you lost some weight,’ so I immediately changed my diet at that point from having been diagnosed to knowing I would have surgery in ten weeks’ time." |  |
|  | “He [the General Practitioner] was a bit skeptical about it [diet] but he has now come around and says, “Well there are some slight indications that it may help.” |  |
| Barriers | “Sometimes I work 10 or 12 hours a day … not much time for anything else.” | ^33,38,39,42,43,45^ |
|  | “Why do I have to change and promote my health if I still don't know if I follow the guidelines or not? The change needs to start from the knowledge on what I must do or not for my cancer.” |  |
|  | “I’ve always run…but as I said, that’s a bit too hard on your body when you get a bit older”. |  |
|  | "I did think if I’d been working, especially over the winter, it would have been quite difficult to do because you get up and go into work in the dark and come home in the dark." |  |
|  | "… I went to my son’s [place] and they don’t eat a lot of fruit and vegetables there right now, so perhaps for a couple of days then, it was a low count.” |  |
|  | “Well, I went to the doctor, and he said, ‘Well, ignore it, don’t worry and don’t bother at all.’ And I said to the doctor, ‘What about diet?’ and he said nothing – "Diet is useless. It doesn’t make a difference. Eat what you like.’ And I took the wrong message. So what I thought the doctor said was do what I wanted to do, which was because I felt that this would make me healthier...It [the General Practitioner’s reaction] didn’t seem to be a healthy living message given and it surprised me that maybe just the attitude of doctors who look for people who are ill and make them better rather than preventative...it didn’t seem to be a priority." |  |
|  | “I want to go to the gym soon, but like I said, because I’m so wet [incontinence] I won’t have – I’m hoping that it will ease up a little bit so I can get to do something else…” |  |
| Lifestyle advice support | | |
| Dietary information for PCa prevention | “Oh very often there are conflicting messages come over but when you realize that we get most of our information from the television we don’t look through scientific journals to uh to weigh up the evidence, and if you go on the internet you just get conflicting stories the worst thing you can do is go on the internet, you don’t know which are the reliable sources.” | ^36,38^ |
|  | “I think a lot of reports such as that are made as a result of very limited studies. Umuh where they have found that uh with a small uh a certain sample of the population um drinking blueberry juice or um before that it was cranberry juice um yeah has a beneficial effect or might have a beneficial effect because of antioxidants in those products. Um but you know there’s uh as far as I know there has been no rigorous scientific studies to prove that it’s effective.” |  |
|  | “I don’t think I would go along with something just because I read it in the paper. If it was if it was information coming from our local GP surgery or one of my visits there then obviously, I would go you know I would bear in mind…would believe that source. I wouldn’t necessarily take it from a newspaper.” |  |
|  | "Yes, dietary advice is available but not specifically for prostate cancer...There are a lot of different sources aren’t there? I mean, there’s a lot. . .published sources are often a lot nowadays.” |  |
| Attitudes Towards Exercise Support | “I think that would be the way to do it, would it, I think. Because then obviously, you're more willing to do it, you have motive to do it. So yeah… yeah that'd definitely be the best way rather than sort of set yourself up, and then you'll just go the first few months and gradually stop.” | ^40,42^ |
|  | “I think I have enough, as I say it's my sort of working knowledge and background and I feel as though I'm sufficiently armed and have enough information to, to enable me to make some good choices.” |  |
|  | “As a Blackman I would want to see some men taught in a way that I can see myself benefitting. I like sports and I can see it incorporated in some type of sport activity. I don’t know exactly how, but I think that’s one way.” |  |
| Exercise Support Preferences | “I know that ah for me personally having something group-based is likely to be much more successful than me doing it myself, that's me.” | ^42^ |
|  | “I think if it's recommended by a specialist, a doctor, a specialist nurse … it would be more likely that somebody would do it.” |  |
|  | “Whoever is doing the ah, the supervising of gym training, should not only know what to do, and ah what it’s doing for you, but also link it to your cancer … if you work on this, it's going to make this a little easier from the point of view of X or Y, that way it is aimed at the disease as well as the man.” |  |
|  | “If you've got something online and you're recommending exercise, you're giving information as to ah what the current treatments are, ah you know any changes in treatment … I think that'd definitely be helpful.” |  |
